# Junction Formation and Leakage Current Suppression in Planar High-Purity Germanium Detectors for Low-Energy X-Ray Detection

**DOI:** 10.3390/ma19143008

**Published:** 2026-07-13

**Authors:** Meng Cao, Qingzhi Hu, Yanggang Jia, Zexin Wang, Zhaoran Guan, Haofei Huang, Linjun Wang, Jian Huang

**Affiliations:** 1 State Key Laboratory of Materials for Advanced Nuclear Energy & School of Materials Science and Engineering, Shanghai University, Shanghai 200444, China; 23722490@shu.edu.cn (Q.H.); ygjia1999@163.com (Y.J.); wangzexin0208@shu.edu.cn (Z.W.); monicazubek@163.com (Z.G.); jianhuang@shu.edu.cn (J.H.); 2 Zhejiang Institute of Advanced Materials, Shanghai University, Jiaxing 314113, China; 3 Shanghai Engineering Research Center for Integrated Circuits and Advanced Display Materials, Shanghai University, Shanghai 200444, China

**Keywords:** high-purity germanium detector, junction regulation, leakage current suppression, X-ray detection

## Abstract

This study addresses the need for dark-current control and stable current response in planar high-purity germanium (HPGe) detectors for low-energy X-ray detection. A device fabrication strategy based on the coupled optimization of near-surface treatment, N/P junction formation, and guard-ring electrode design is proposed. Unlike previous studies that mainly focused on contact-layer fabrication, segmented electrode structures, low-noise readout, or response simulation, this work investigates low-damage near-surface construction, N-type and P-type contact-layer formation, and edge-related leakage-current regulation as an interconnected processing route. The relationship among the near-surface state, junction quality, electrode configuration, and edge-related leakage current is emphasized. Chemical mechanical polishing (CMP) reduced the surface roughness Sa of the HPGe crystal to 6.68 nm, providing a low-damage near-surface foundation for subsequent junction fabrication. On this basis, the optimized Li thermal diffusion process, namely 0.5 Å s^−1^, 325 °C, and 5 min, formed an N-type contact layer with preserved lattice ordering and favorable electrical properties. B ion implantation combined with rapid thermal processing (RTP) achieved acceptor activation and implantation-damage recovery, and the condition with Rp = 198.1 nm showed relatively better structural recovery and electrical characteristics. After introducing the guard-ring electrode, the dark current of the device at −20 V decreased from 6.5 × 10^−9^ A to 2.03 × 10^−9^ A, and a stable switching current response was obtained under 12 keV monochromatic synchrotron X-ray irradiation. Geant4 simulations were further used as an auxiliary analysis to evaluate the effect of the guard-ring structure on the simulated response spectra and full-energy peak efficiency (FEPE) for low-energy X-rays. Overall, this study provides experimental evidence for process optimization of planar HPGe detectors with low dark current and stable low-energy current response.

## 1. Introduction

High-purity germanium (HPGe) detectors possess distinct advantages in high-resolution X/γ-ray spectroscopy owing to the relatively high atomic number of Ge, the low average energy required for electron–hole pair creation, and the small Fano factor [1,2,3,4]. In recent years, with the development of low-background measurements, precision nuclear spectroscopy, and low-energy radiation detection, higher requirements have been imposed on HPGe detectors in terms of low leakage current, low detection threshold, and stable low-energy response [5,6,7,8]. However, HPGe is a narrow-bandgap semiconductor and generally requires operation at cryogenic temperatures. Even near 77 K, factors such as surface states, interfacial defects, contact-layer quality, and dead-layer thickness can still significantly affect the dark current and low-energy detection performance of the device [9,10,11,12].

Compared with coaxial and large-volume configurations, planar HPGe detectors have a simpler structure, a thinner front window, and lower parasitic capacitance, making them more suitable for detecting low-energy X-rays and low-energy γ-rays. They also provide a basis for the design of segmented electrodes, small-area readout, and position-sensitive structures [13,14,15]. Previous studies have explored planar or related advanced HPGe detectors in terms of contact-layer fabrication, segmented structures, low-noise readout, and response simulation [13,14,15,16,17]. However, these studies have mostly focused on individual aspects, such as single-side contact-layer preparation, electrode-structure design, readout-noise control, or detection-efficiency calibration. For planar HPGe detectors, the near-surface state, N/P junction quality, and edge-related surface leakage current are not independent issues. Instead, they are key factors that jointly affect the dark current and the current-response stability under low-energy X-ray irradiation [16,17,18,19,20]. Near-surface processing damage, surface roughness, and surface oxide states can affect doping uniformity, interfacial stability, and contact-layer quality during subsequent Li thermal diffusion and B ion implantation. At the same time, the electrode structure can modify the potential distribution near the sidewall and edge regions, making the surface leakage current more or less likely to couple into the central readout electrode. Therefore, changes in the dark current and low-energy X-ray response stability of planar HPGe detectors cannot be fully explained by considering only a single surface-treatment process, a single doping parameter, or a single electrode structure.

Based on these considerations, this work first investigated the surface planarization process of chemical–mechanical polishing (CMP) and used it as a low-damage near-surface foundation for subsequent Li thermal diffusion and B ion implantation. The effects of surface roughness, chemical state, and lattice integrity on junction fabrication were analyzed. Second, the Li thermal diffusion process for the N-type contact layer and the B ion implantation process for the P-type contact layer were optimized separately. Raman spectroscopy, X-ray photoelectron spectroscopy (XPS), Hall measurements, secondary ion mass spectrometry (SIMS), and Kelvin probe force microscopy (KPFM) were then used to establish the relationship among junction structure, electrical properties, and in-plane uniformity. Finally, after the N/P junction was constructed, a guard-ring electrode structure was introduced to further analyze the effect of electrode configuration on edge-related leakage current and the dark current of the central readout electrode. Through the coupled design of near-surface treatment, N/P junction formation, and guard-ring electrode structure, this study aims to establish the relationship among the near-surface state, junction quality, electrode configuration, and leakage-current behavior. Low-temperature dark-current measurements and synchrotron X-ray current-response measurements were used as the main device-level validation methods, providing experimental evidence for optimizing dark-current suppression and low-energy response stability in planar HPGe detectors.

## 2. Materials and Methods

### 2.1. HPGe Crystal Pretreatment and CMP Process

The HPGe crystals used in this study were purchased from Umicore (Brussels, Belgium), with original dimensions of Φ50.8 mm × 1 mm or Φ50.8 mm × 2 mm. The net impurity concentration was on the order of 10^10^ cm^−3^, and the crystal orientation was <100>. The crystals were cut into planar device samples with dimensions of 10 mm × 10 mm × 1 mm using a diamond-wire saw (STX-402, Shenyang Kejing Auto-Instrument Co., Ltd., Shenyang, China), followed by sequential ultrasonic cleaning in acetone (Sinopharm Chemical Reagent Co., Ltd., Shanghai, China), ethanol (Sinopharm Chemical Reagent Co., Ltd., Shanghai, China), and deionized water produced using a UPTA ultrapure water system (Lichen Scientific Instrument (Zhejiang) Co., Ltd., Shaoxing, China).

During the mechanical polishing (MP) stage, a UNIPOL-820 grinding and polishing machine (Shenyang Kejing Auto-Instrument Co., Ltd., Shenyang, China) was used. The rotation speed of the polishing plate, polishing time, and polishing-pad type were selected as the main variables. The rotation speeds were set to 80 and 100 r min^−1^, the polishing times were set to 30 and 60 min, and the polishing pads included suede and polyurethane pads (Shenyang Kejing Auto-Instrument Co., Ltd., Shenyang, China). After MP, chemical–mechanical polishing (CMP) was performed using a 0.05 μm SiO_2_ suspension (Shenyang Kejing Auto-Instrument Co., Ltd., Shenyang, China) containing 0.5 wt% H_2_O_2_ (Sinopharm Chemical Reagent Co., Ltd., Shanghai, China) and 0.24 M C_6_H_8_O_7_ (Sinopharm Chemical Reagent Co., Ltd., Shanghai, China) as the polishing system. The CMP rotation speed was 60 r min^−1^, and the polishing time was 5 min.

### 2.2. Li Thermal Diffusion and B Ion Implantation

The purity of the Li source was 99.95%, and the Li source was purchased from Dongguan Canrd Laboratory Equipment Technology Co., Ltd. (Dongguan, China). Li thermal diffusion was carried out in an integrated thermal evaporation and glovebox system (VZZ-300S, Beijing MicroNano Vacuum Technology Co., Ltd., Beijing, China) to prevent oxidation of metallic Li in air. First, the effects of different Li deposition rates on the near-surface structure of HPGe were compared. Subsequently, at a deposition rate of 0.5 Å s^−1^ and a diffusion time of 5 min, the diffusion temperatures were set to 300, 325, and 350 °C to determine the suitable process window for N-region fabrication.

For B ion implantation, three implantation conditions were first simulated using Sentaurus TCAD X-2025.09 (Synopsys, Inc., Sunnyvale, CA, USA): 30 keV, 1 × 10^14^ cm^−2^; 50 keV, 3 × 10^14^ cm^−2^; and 70 keV, 4 × 10^14^ cm^−2^. The implantation tilt angle was fixed at 7° for all conditions. The projected range Rp was calculated from the simulated concentration profiles, and the corresponding Rp values for the three conditions were 93.3, 198.1, and 258.8 nm, respectively. B ion implantation was performed using a Danfysik 1080-30 high-precision ion implantation system (Danfysik A/S, Taastrup, Denmark) at Beijing Zhongjian Weikang Electronic Technology Co., Ltd. (Beijing, China). After implantation, the samples were subjected to rapid thermal annealing using an RTP-300 rapid thermal processing system (Beijing East Star Institute of Applied Physics, Beijing, China) at 450 °C for 60 s to repair implantation-induced damage and promote acceptor activation.

### 2.3. Structural, Chemical-State, and Electrical Characterization

The surface and cross-sectional morphologies of the samples were observed using field-emission scanning electron microscopy (FE-SEM, Sirion 200, FEI, Hillsboro, OR, USA). Energy-dispersive X-ray spectroscopy (EDS) was used to analyze the surface elemental distribution and possible residues after CMP. The three-dimensional surface morphology and roughness of the samples were measured using a 3D optical profiler (Sensofar, Barcelona, Spain). The roughness parameters included the arithmetic mean height (Sa), maximum peak-to-valley height (PV), and full width at half maximum (FWHM) of the height distribution. The surface chemical states were characterized by X-ray photoelectron spectroscopy (XPS, ESCALAB 250Xi, Thermo Scientific, Waltham, MA, USA). This system enables high-resolution analysis of surface elements and their chemical states, typically using a monochromatic Al Kα X-ray source. The crystal structure was characterized using Raman spectroscopy (JY H800UV, HORIBA Jobin Yvon S.A.S., Palaiseau, France), which was employed to analyze the near-surface lattice ordering and local structural perturbations after CMP treatment, Li thermal diffusion, and B ion implantation. A 532 nm excitation laser was used for the Raman measurements. Low-temperature photoluminescence (PL) measurements at 77 K were performed using a fluorescence spectrometer system (FLS980, Edinburgh Instruments Ltd., Livingston, UK) to investigate near-surface radiative recombination behavior and defect-related emission changes. The depth distribution of implanted B was measured by secondary ion mass spectrometry (SIMS, Kore SurfaceSeer-I, Ely, UK). The surface potential distribution was characterized using Kelvin probe force microscopy (KPFM, Bruker Dimension Icon, Berlin, Germany) to evaluate the local electrical uniformity of the doped layers. The carrier type, concentration, resistivity, and mobility were obtained using a Hall measurement system (HMS-5000, Ecopia Corporation, Anyang, Republic of Korea) to assess the electrical properties of the Li-diffused and B-implanted layers.

### 2.4. Device Fabrication, Cryogenic Electrical Measurements, Synchrotron X-Ray Current-Response Measurements, and Simulation Analysis

**Figure 1:**
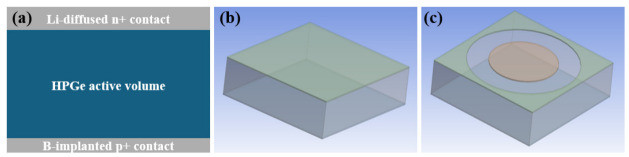
Schematic illustrations of the planar HPGe detector junction structure and the two fabricated electrode configurations: (**a**) Cross-sectional schematic of the planar HPGe detector, showing the Li-diffused n+ contact, HPGe active volume, and B-implanted p+ contact. (**b**) Schematic of the full-coverage electrode structure. (**c**) Schematic of the guard-ring electrode structure.

As shown in Figure 1, the basic N/P junction structure of the planar device was first constructed on the CMP-treated HPGe crystal. One side was processed by Li thermal diffusion to form the Li-diffused n^+^ contact, while the opposite side was processed by B ion implantation combined with rapid thermal processing (RTP) to form the B-implanted p^+^ contact. The middle region served as the HPGe active volume. On this basis, two device configurations were further fabricated, namely the full-coverage electrode structure shown in Figure 1b and the guard-ring electrode structure shown in Figure 1c. For the full-coverage electrode structure, the top electrode continuously covers the device surface and extends to the edge region. Therefore, edge-related surface leakage current may more easily couple into the readout electrode. For the guard-ring electrode structure, an independent guard-ring electrode was arranged around the central readout electrode to regulate the current path near the edge region and reduce the influence of edge-related surface leakage current on the central readout electrode.

The electrodes were prepared using a high-vacuum resistance evaporation coating system (VZZ-300S, Beijing MicroNano Vacuum Technology Co., Ltd., Beijing, China). A 10 nm Ni layer and a 50 nm Au layer were sequentially deposited on the N-type region, while a 10 nm Pd layer and a 50 nm Au layer were sequentially deposited on the P-type region. The Ni, Pd, and Au evaporation sources, each with a purity of 99.999% (5N), were purchased from Zhongke Yannuo (Beijing) Technology Co., Ltd. (Beijing, China), Materion Corporation (Cleveland, OH, USA), and Umicore Metal Deposition Solutions (Brussels, Belgium), respectively. During deposition, the vacuum in the chamber was maintained at approximately 2 × 10^−4^ Pa, and the substrate holder was rotated at 15 rpm to improve the uniformity of the electrode films. For Ni deposition, the evaporation current was approximately 135.9 A, and the deposition rate was maintained at 0.3 Å s^−1^ until the thickness reached 10 nm. For Pd deposition, the evaporation current was approximately 155.4 A, and the deposition rate was maintained at 0.3 Å s^−1^ until the thickness reached 10 nm. The Au layers on the N-type and P-type regions were deposited under the same conditions, with an evaporation current of approximately 96.6 A and a deposition rate of 0.3 Å s^−1^ until the thickness reached 50 nm. The full-coverage electrode structure and the guard-ring electrode structure were fabricated using the same Li-diffused n^+^ contact, B-implanted p+ contact, metal electrode system, and electrode-deposition process, so as to minimize the influence of differences in junction fabrication and metallization processes on the comparison of dark-current characteristics.

The dark current of the devices was measured at 77 K using a cryogenic probe station (TTPX cryogenic probe station, Lake Shore Cryotronics, Westerville, OH, USA), which is suitable for low-temperature electrical measurements under liquid-nitrogen or liquid-helium environments. The X-ray response current of the devices was measured at the BL15U1 beamline of the Shanghai Synchrotron Radiation Facility. BL15U1 is a hard X-ray microfocus beamline that provides an energy-tunable, high-flux monochromatic X-ray microbeam with an energy range of approximately 5–20 keV, making it suitable for micro-area X-ray fluorescence, absorption, and diffraction experiments. The potential distribution of the devices was simulated using Ansys Lumerical CHARGE 2025 R2 (ANSYS, Inc., Canonsburg, PA, USA) to compare the effects of different electrode structures on the edge electric field and surface current collection paths. Geant4 was used as an auxiliary tool to analyze the simulated response spectra and full-energy peak efficiency (FEPE) for low-energy X-rays. Specifically, Geant4 version 11.0.0 was used to compare the simulated response spectra and FEPE of ^55^Fe 5.9 keV low-energy X-rays for the full-coverage electrode structure and the guard-ring electrode structure. It should be noted that a complete detector-response model was not established in this work. Leakage-current noise, front-end electronic noise, charge trapping, carrier-transport loss, and readout-circuit response were not quantitatively included in the Geant4 simulation. Therefore, the simulated spectral width obtained from Geant4 cannot be used as the actual energy resolution. It can only serve as an auxiliary result for comparing the simulated response spectra and FEPE variations between different electrode structures.

## 3. Results and Discussion

The leakage current and low-energy X-ray current response of planar HPGe detectors are jointly affected by the near-surface state, N/P junction quality, and electrode configuration. Since both Li thermal diffusion and B ion implantation occur in the near-surface region, residual processing damage, surface fluctuation, and unstable surface oxides may affect doping uniformity, contact-layer formation, interfacial stability, and subsequent leakage-current behavior. Therefore, before evaluating the electrical characteristics and low-energy X-ray response of the device, it is necessary to first establish a near-surface foundation with low roughness, low damage, and relatively controllable chemical states. Based on this logic, this section sequentially analyzes the relationship among near-surface treatment, N/P junction formation, guard-ring electrode regulation, and low-energy X-ray response in order to clarify their roles in leakage-current suppression and response stability in planar HPGe detectors.

### 3.1. Construction of a Low-Damage Near-Surface Layer

**Figure 2:**
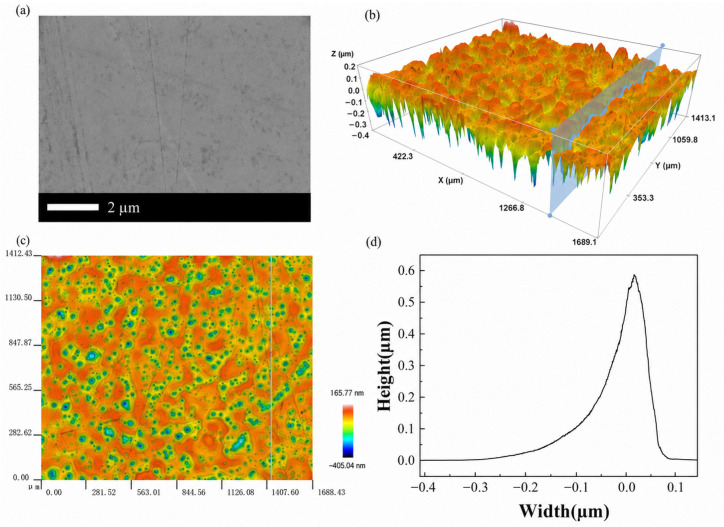
Surface morphology of the HPGe crystal polished under the condition of 100 r min^−1^–30 min–polyurethane: (**a**) SEM image; (**b**) 3D topographic map and height distribution; (**c**) 2D surface map; (**d**) roughness distribution histogram.

The complete SEM and 3D profilometry results of HPGe crystal surfaces under different MP conditions are shown in Supplementary Figures S1–S7, and the statistical results obtained from 3D profilometry are summarized in Table 1. Overall, the polishing performance of the polyurethane pad was significantly better than that of the suede pad. A moderate increase in rotation speed was beneficial for improving surface flatness, whereas further prolonging the polishing time provided only limited improvement. As shown in Figure 2, under the condition of 100 r min^−1^, 30 min, and a polyurethane pad, only a small number of shallow fine scratches and dispersed micro-defects remained on the sample surface, while no obvious blocky spalling or large-scale grooves were observed. This indicates that MP under this condition can sufficiently remove the major surface damage. The corresponding 3D profilometry parameters Sa, PV, and FWHM were 47.87 nm, 314.72 nm, and 77.06 nm, respectively, all of which were markedly better than those obtained using the suede pad. Considering both the improvement in surface morphology and process efficiency, 100 r min^−1^, 30 min, and a polyurethane pad can be recommended as the MP pretreatment parameters for subsequent CMP processing.

**Figure 3:**
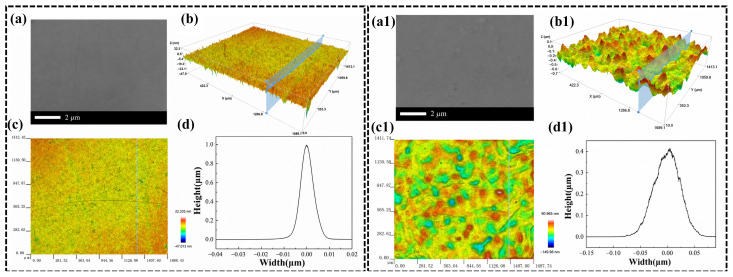
Surface morphology of the CMP-treated HPGe crystal: (**a**) SEM image; (**b**) 3D topographic map and height distribution; (**c**) 2D surface map; (**d**) roughness distribution histogram. Surface morphology of the chemically etched HPGe crystal: (**a1**) SEM image; (**b1**) 3D topographic map and height distribution; (**c1**) 2D surface map; (**d1**) roughness distribution histogram.

As shown in Figure 3, after CMP treatment, the HPGe surface became more uniform overall. The stripe-like textures, local deep grooves, and sharp protrusions remaining after MP were significantly weakened, with only a few shallow fine scratches retained. No obvious blocky spalling or large-scale etch pits were observed, indicating that CMP can further reduce surface irregularities under relatively mild processing conditions. Combined with the MP results discussed above, the role of CMP is mainly reflected in two aspects: reducing residual peak-valley fluctuations and improving surface uniformity. This is consistent with the reported CMP mechanism for brittle semiconductor materials, in which low-damage surface processing can be achieved through surface oxidation and removal of the softened surface layer [21]. The corresponding 3D profilometry results show that the Sa, PV, and FWHM of the CMP-treated sample decreased to 6.68 nm, 25.43 nm, and 6.32 nm, respectively, indicating that the surface roughness was further reduced from the tens-of-nanometers level after MP to the single-digit nanometer range. In conventional HPGe detector fabrication, mixed-acid chemical etching using HNO_3_ and HF is commonly performed after MP to improve surface quality. Meng et al. reported an etching solution ratio of 4:1 concentrated HNO_3_/HF for HPGe detector fabrication, which was used to remove surface pits and scratches and obtain a relatively bright surface [22]. However, compared with CMP, the HF-HNO_3_ chemically etched sample still exhibited obvious etching textures and corrosion pits, with Sa, PV, and FWHM values of 19.14 nm, 103.35 nm, and 57.05 nm, respectively. This indicates that CMP not only further reduces the average surface roughness but also more effectively suppresses local deep valleys and sharp peaks while significantly narrowing the surface height distribution. Based on the SEM and 3D profilometry results, CMP showed better surface planarization and local damage suppression than conventional chemical etching under the present experimental conditions and is therefore more suitable as a surface pretreatment step for subsequent junction-formation processes.

To verify whether CMP introduced surface residues or structural damage, the samples were characterized by EDS, XPS, Raman spectroscopy, and low-temperature PL at 77 K, as shown in Supplementary Figures S8–S11. The results show that the surface of the CMP-treated sample remained dominated by Ge, with an O content of only 0.86%, and no obvious impurity elements were detected, indicating that residues from the polishing slurry and etchants were negligible. Regarding the trace amount of O, previous studies have shown that Ge can be rapidly reoxidized in air after wet processing, and surface Ge oxides can reform within a short time [23]. Therefore, the small amount of detected O mainly originated from the surface oxide layer formed after the polished sample was exposed to air. The XPS results show that the CMP-treated HPGe surface was still dominated by the intrinsic Ge chemical state, with only a small amount of Ge oxide present in the outermost surface layer. The Raman spectrum exhibited the characteristic crystalline Ge peak at 300.55 cm^−1^, without any obvious amorphization signal. The 77 K low-temperature PL spectrum retained the main emission peak at 0.7209 eV, corresponding to the intrinsic indirect bandgap emission of Ge. These results indicate that CMP improved the surface quality while preserving the main lattice characteristics of HPGe within the detection limits of the present measurements.

Based on the above results, the subsequent Li thermal diffusion and B ion implantation processes were both performed on CMP-treated HPGe surfaces. In other words, the role of CMP in this work is not an isolated optimization of the surface process but rather to provide a near-surface foundation with low roughness, low damage, and controllable chemical states for subsequent junction formation. This helps reduce the interference of pretreatment-induced defects with doping uniformity and device electrical performance.

### 3.2. Coupled Construction of Li and B Junction Regions

#### 3.2.1. Li Thermal Diffusion for Constructing the N-Type Contact Layer

To avoid near-surface disordering and excessive lithiation caused by an overly high Li incorporation amount, the Li evaporation rate was first prescreened. The results show that a higher Li deposition rate led to broadening or even disappearance of the characteristic crystalline Ge peak, accompanied by the appearance of low-binding-energy Ge-related components, indicating reduced structural stability in the near-surface region. Therefore, the subsequent temperature optimization was performed at a Li deposition rate of 0.5 Å s^−1^, and the related results are provided in Supplementary Figures S12 and S13.

**Figure 4:**
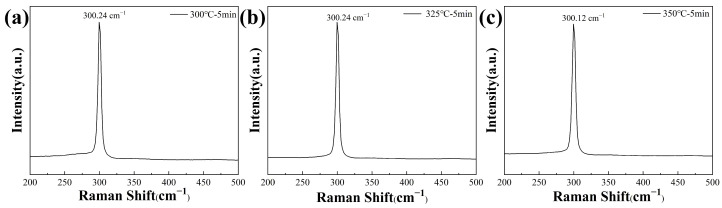
Raman spectra of HPGe crystals at different Li diffusion temperatures: (**a**) 300 °C; (**b**) 325 °C; (**c**) 350 °C.

Under a fixed Li evaporation rate of 0.5 Å s^−1^ and a diffusion time of 5 min, the effect of diffusion temperature on the formation of the N-type contact layer was further investigated. The Raman spectra obtained at different diffusion temperatures are shown in Figure 4. The samples treated at 300, 325, and 350 °C all retained the first-order optical phonon peak of crystalline Ge near 300 cm^−1^. However, the 325 °C sample exhibited the smallest peak width, with a full width at half maximum (FWHM) of 4.75 cm^−1^. In comparison, the FWHM values of the 300 and 350 °C samples increased to 4.992 and 5.53 cm^−1^, respectively. This indicates that the 325 °C condition better preserved the lattice ordering and reduced local structural perturbation induced by Li diffusion compared with the other tested temperatures. In general, a narrower Raman peak indicates higher lattice ordering and weaker defect- and strain-related disorder, whereas peak broadening is usually associated with increased structural disorder. Based on the experimental results, within the temperature range of 300 to 350 °C, the 325 °C sample exhibited the narrowest Raman peak and a relatively stable peak position. This suggests that the lattice ordering was best preserved and that the influence of Li diffusion on the HPGe lattice was minimized under this condition [24,25,26]. In addition to Raman analysis, XPS was performed to examine the surface chemical states of the Li-diffused HPGe samples, and the results are shown in Supplementary Figure S14. The survey spectra indicate that the sample surfaces were dominated by Ge signals, accompanied by surface O and C signals and a weak Li-related signal. The high-resolution Ge 3d spectra were still mainly composed of the elemental Ge doublet. Only minor oxidized Ge components were observed, which can be attributed to the thin native oxide layer formed after air exposure. The Li 1s signal was detected for all Li-diffused samples, confirming the incorporation of Li within the XPS probing depth. Among the three diffusion temperatures, the 325 °C sample maintained a well-defined Ge 3d line shape and a detectable Li 1s signal. This indicates that Li introduction under this condition did not induce severe surface chemical disorder. Considering the surface sensitivity of XPS and the relatively weak Li 1s intensity, the XPS results are used here as complementary evidence for Li incorporation and surface chemical stability. The optimization of the Li diffusion condition is mainly based on the combined Raman and Hall results.

The room-temperature Hall measurement results further support this conclusion, as shown in Table 2. All three samples exhibited N-type conductivity, indicating that donor-type electrically active centers were formed in the near-surface region of HPGe after Li thermal diffusion. Compared with the samples treated at 300 and 350 °C, the 325 °C sample exhibited the lowest resistivity of 37.50 Ω cm and the highest mobility of 1166 cm^2^ V^−1^ s^−1^. This indicates more sufficient activation of Li donors, weaker defect scattering, and lower local structural perturbation under this condition. The 300 °C sample showed relatively low carrier concentration and electrical conductivity, suggesting insufficient diffusion and donor activation. Although the carrier concentration further increased in the 350 °C sample, its mobility decreased significantly. This indicates that ionized-impurity scattering, local strain, and defect-related scattering began to intensify under an excessive thermal budget [24,27,28,29]. In addition, the Li-diffused n^+^ layer usually contains a dead layer and a semi-active transition layer, where charge collection is incomplete. This may introduce slow pulses and energy-degradation events in the low-energy region. Therefore, the selection of diffusion temperature is related not only to donor activation efficiency but also to the subsequent low-energy spectroscopic performance and operational stability of the device [24]. Based on the Raman, XPS, and Hall results, 0.5 Å s^−1^, 325 °C, and 5 min are identified as the optimized process conditions for constructing the N-type contact layer by Li thermal diffusion in this work.

**Figure 5:**
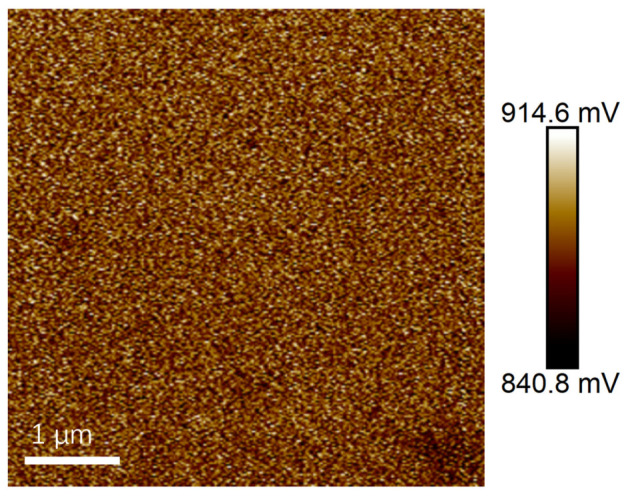
KPFM image of HPGe after thermal diffusion doping.

To further evaluate the in-plane uniformity of the Li-diffused layer under the optimized condition, KPFM characterization was performed on the sample treated at 0.5 Å s^−1^, 325 °C, and 5 min, as shown in Figure 5. The surface potential distribution of the sample was overall uniform, with an average surface potential of 0.878 V and a standard deviation of only 0.0105 V. This relatively small potential fluctuation suggests good lateral electrical uniformity of the Li-diffused layer on the micrometer scale. KPFM is commonly used to characterize the surface work function and local potential fluctuations of semiconductors [30,31,32,33]. Combined with the structural and transport results discussed above, it can be concluded that the optimized Li thermal diffusion process is capable of forming an N-type contact layer with good structural integrity, uniform distribution, and favorable electrical properties on the low-damage near-surface foundation established by CMP.

#### 3.2.2. B Ion Implantation for Constructing the P-Type Contact Layer

**Figure 6:**
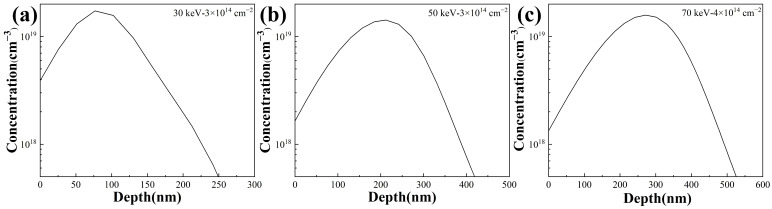
Ion implantation simulations: (**a**) 30 keV, 1 × 10^14^ cm^−2^; (**b**) 50 keV, 3 × 10^14^ cm^−2^; (**c**) 70 keV, 4 × 10^14^ cm^−2^.

To construct a stable P-type contact layer on the opposite side of the HPGe crystal, Sentaurus TCAD was first used to simulate the B ion implantation process and determine the projected range (Rp) under different implantation conditions. As shown in Figure 6, the Rp values corresponding to 30 keV, 1 × 10^14^ cm^−2^; 50 keV, 3 × 10^14^ cm^−2^; and 70 keV, 4 × 10^14^ cm^−2^ were 93.3, 198.1, and 258.8 nm, respectively. Rp was used to characterize the implantation depth because it reflects the average stopping position of ions along the depth direction and is therefore more suitable for comparing the distribution characteristics of implanted layers under different implantation conditions [34,35].

**Figure 7:**
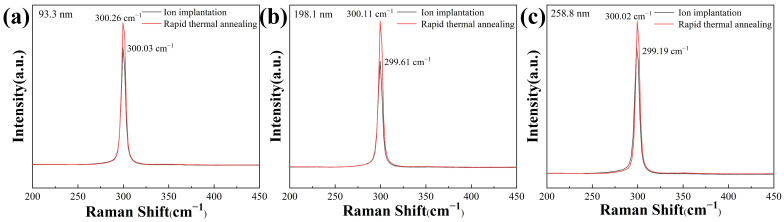
Raman spectra of HPGe crystals with different implantation depths: (**a**) 93.3 nm; (**b**) 198.1 nm; (**c**) 258.8 nm.

The Raman results of samples with different Rp values before and after RTP are shown in Figure 7. As the implantation depth increased from 93.3 to 258.8 nm, the Raman peak of the as-implanted samples gradually shifted to lower wavenumbers, and the peak width increased from 5.27 cm^−1^ to 5.83 cm^−1^, indicating that B implantation with higher energy and dose introduced stronger lattice perturbation and disorder. This trend is consistent with the general behavior of damage accumulation and amorphization in Ge during ion implantation [34,35,36]. After RTP treatment, the peak positions of all groups recovered to around 300 cm^−1^, and the peak widths became significantly narrower, indicating that the annealing process effectively repaired the lattice damage induced by ion implantation and improved the crystal ordering. Notably, for the 198.1 nm group, the Raman peak position after RTP recovered to 300.11 cm^−1^, and the FWHM decreased to 4.94 cm^−1^, which was close to that of the 93.3 nm group. In contrast, although the 258.8 nm group also showed a certain degree of recovery, its residual peak width remained relatively large, indicating that more pronounced lattice perturbation was still retained under the deeper implantation condition. Therefore, from the perspective of balancing effective acceptor incorporation and damage recoverability, the condition corresponding to Rp = 198.1 nm is more suitable for constructing the P-type contact layer.

The room-temperature Hall measurement results further support the above conclusion, as shown in Table 3. All three samples exhibited P-type conductivity, indicating that acceptor-type electrically active layers were successfully formed after B ion implantation followed by RTP. As Rp increased from 93.3 to 198.1 nm, both the bulk carrier concentration and sheet carrier concentration increased significantly, while the resistivity decreased, indicating enhanced effective activation of B acceptors. When Rp was further increased to 258.8 nm, although the implantation became deeper, the carrier concentration and electrical conductivity instead decreased. This suggests that defect compensation and carrier trapping induced by the higher implantation energy and dose began to offset the acceptor activation effect. Among these samples, the 198.1 nm group exhibited the highest bulk carrier concentration (8.52 × 10^15^ cm^−3^), the highest sheet carrier concentration (4.26 × 10^14^ cm^−2^), and the lowest resistivity (7.69 Ω·cm), suggesting a more favorable balance between B acceptor activation and implantation-damage control was achieved under this condition [37].

**Figure 8:**
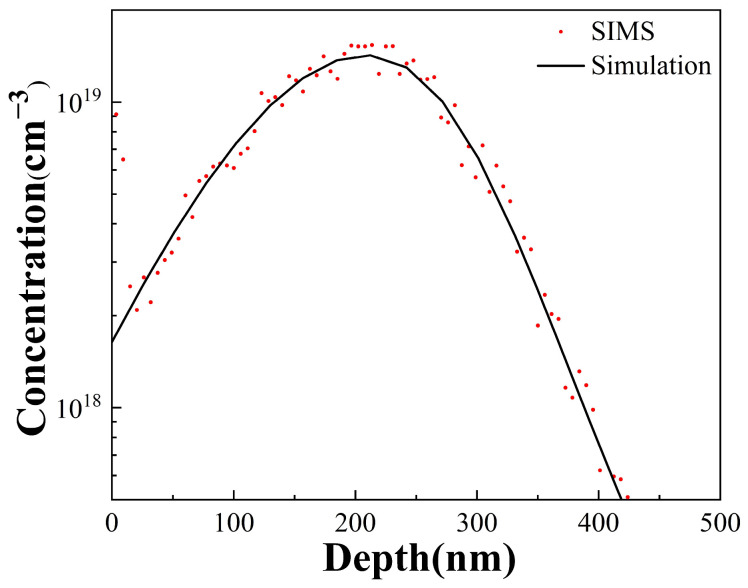
Comparison between the simulated and SIMS-measured B profiles in the HPGe crystal after B ion implantation.

To verify the reliability of the simulation results, SIMS measurements were performed on the optimized sample with Rp = 198.1 nm, as shown in Figure 8. Within a depth range of approximately 400 nm, the experimentally measured B distribution agreed well with the TCAD-simulated profile, indicating that the simulation can reasonably predict the implantation depth and distribution trend. Therefore, the 198.1 nm condition not only exhibits the best performance in terms of structural recovery and electrical properties but also has an experimentally validated implantation profile. Thus, it can be used as the process parameter for constructing the P-type contact layer in subsequent device fabrication. Further XPS, KPFM, and low-temperature PL results at 77 K are provided in Supplementary Figures S15–S19. These results collectively indicate that the 198.1 nm sample exhibits good near-surface uniformity after RTP and forms an electrically active doped layer dominated by B-related shallow acceptor centers.

### 3.3. Effect of the Guard-Ring Electrode Structure on Dark-Current Behavior

**Figure 9:**
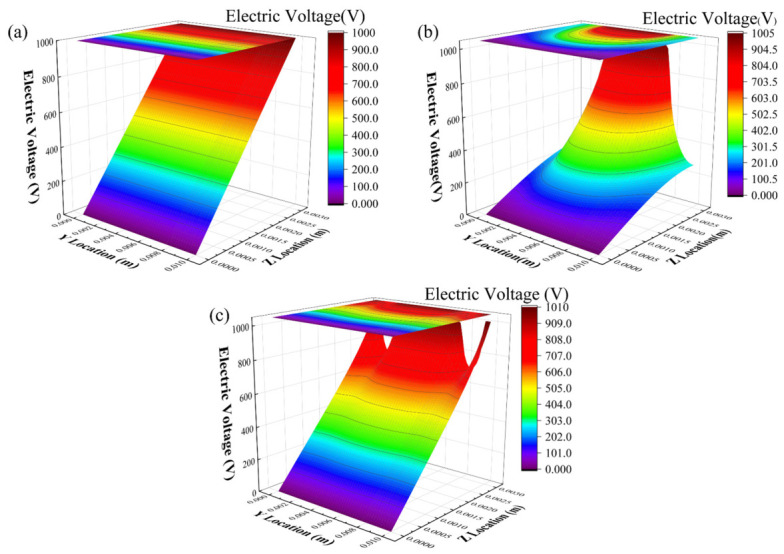
Three-dimensional potential distributions of the electrode structures of HPGe detectors: (**a**) full-coverage electrode; (**b**) point electrode; (**c**) guard-ring electrode.

After determining the optimized process parameters for Li thermal diffusion and B ion implantation, the influence of electrode configuration on the dark-current behavior of planar HPGe detectors was further investigated. In the full-coverage electrode structure, the top electrode continuously extends toward the edge region. When defects, adsorbed layers, or local conductive paths exist near the sidewall surface, edge- and sidewall-related leakage may more easily contribute to the current measured at the readout electrode. In contrast, the guard-ring electrode introduces an annular electrode region outside the central readout electrode, which can regulate the potential distribution near the top-surface edge region and reduce the coupling of edge-related leakage paths to the central readout electrode [38]. The Ansys-simulated potential distributions are shown in Figure 9. Compared with the full-coverage electrode structure, the guard-ring electrode structure forms an annular potential-regulation region around the central readout electrode and redistributes the potential near the top surface and edge region. This potential distribution provides a structural basis for reducing the contribution of edge- and sidewall-related leakage to the central readout current.

**Figure 10:**
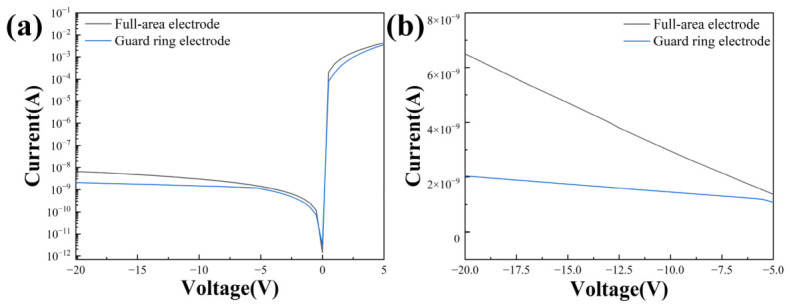
Dark-current characteristics of HPGe detectors with different electrode structures: (**a**) overall view; (**b**) enlarged view.

The low-temperature dark-current characteristics of the devices with full-coverage and guard-ring electrodes were compared under the optimized junction-fabrication conditions, as shown in Figure 10. The dark current of the guard-ring electrode device was consistently lower than that of the full-coverage electrode device over the reverse-bias range. At −20 V, the dark current decreased from 6.5 × 10^−9^ A for the full-coverage electrode device to 2.03 × 10^−9^ A for the guard-ring electrode device. In addition, the current–voltage curve of the guard-ring device increased more gradually with reverse bias, whereas the full-coverage electrode device showed a more rapid increase in dark current. These results indicate that the guard-ring electrode structure reduces the central-electrode dark current and weakens the voltage-dependent increase in leakage current. Combined with the Ansys-simulated potential distribution, the decrease in the central-electrode dark current is consistent with the expected role of the guard-ring electrode in reducing the contribution of edge- and sidewall-related leakage to the central readout current.

### 3.4. Low-Energy X-Ray Current-Response Measurement and Simulation Analysis

**Figure 11:**
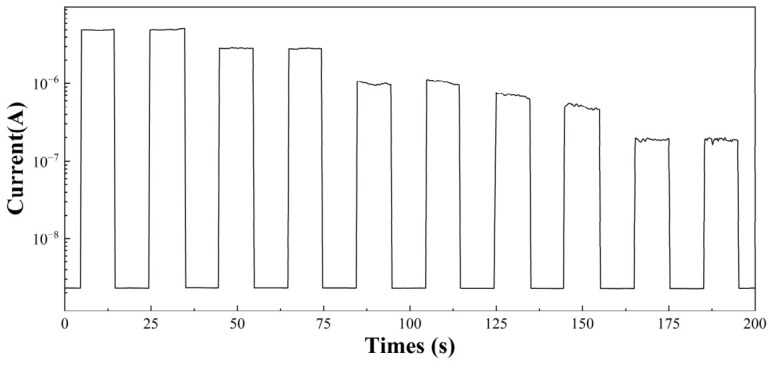
Current–time (I–T) curves of the HPGe detector under different incident photon numbers.

To evaluate the operational stability of the optimized device under low-energy X-ray irradiation, current-response measurements were further performed using 12 keV monochromatic synchrotron X-rays. As shown in Figure 11, during the periodic switching of the X-ray beam on and off, the response current of the device switched stably with the change in the incident photon number. The repeated measurements showed good consistency, and the current rapidly returned to the dark-current level after the X-ray beam was turned off. These results indicate that, after near-surface optimization, N/P junction formation, and guard-ring electrode regulation, the planar HPGe detector exhibits a stable and repeatable current response to 12 keV low-energy X-rays.

**Figure 12:**
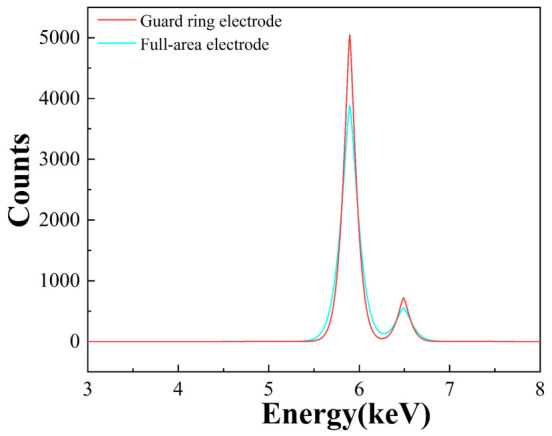
Geant4-simulated response spectra of ^55^Fe low-energy X-rays in the planar HPGe detector.

To further analyze the effect of the guard-ring structure on the simulated low-energy X-ray response and FEPE, Geant4 was used to simulate the response spectra of 5.9 keV X-rays emitted from an ^55^Fe source in the planar HPGe detector [15,19,20]. As shown in Figure 12, the absolute FEPE values of the full-coverage electrode structure and the guard-ring electrode structure were 4.54% and 4.52%, respectively. The difference between the two values is small, suggesting that the isolation region introduced by the guard-ring structure has a limited effect on the simulated effective detection area and FEPE for ^55^Fe 5.9 keV X-rays. Combined with the cryogenic dark-current results in Figure 10 and the synchrotron X-ray current-response results in Figure 11, the guard-ring electrode structure can reduce the dark current of the central readout electrode while maintaining a nearly unchanged FEPE for ^55^Fe 5.9 keV low-energy X-rays. It also allows the device to maintain a stable and repeatable current response under 12 keV low-energy X-ray irradiation. It should be noted that the Geant4 simulation was mainly used to analyze the effect of geometric structure on energy deposition and FEPE. The spectral width in the simulated response spectra cannot be directly regarded as the experimentally measured energy resolution. The actual energy resolution, peak-to-background ratio, charge-collection efficiency, and spectral stability still need to be further evaluated using a cryogenic low-noise spectroscopic readout system.

Taken together, dark-current suppression and low-energy X-ray current-response stability in planar HPGe detectors are jointly affected by near-surface quality, N/P junction quality, and electrode configuration. CMP reduced the surface roughness to 6.68 nm and maintained good lattice integrity and a Ge-dominated surface chemical state, providing a low-damage near-surface foundation for subsequent Li thermal diffusion and B ion implantation. The optimized Li-diffused n^+^ contact and B-implanted p^+^ contact showed good structural recovery, electrical properties, and in-plane uniformity, which are beneficial for forming a relatively stable N/P junction. On this basis, after introducing the guard-ring electrode structure, the dark current of the central readout electrode at −20 V decreased from 6.5 × 10^−9^ A to 2.03 × 10^−9^ A. The reduced dark current lowered the background-current contribution in the low-energy X-ray current response, allowing the device to exhibit a stable and repeatable response under periodic 12 keV synchrotron X-ray irradiation. These results indicate that near-surface treatment, N/P junction formation, and electrode-configuration regulation are not independent factors. Instead, they jointly affect the dark-current behavior and low-energy X-ray current-response stability of planar HPGe detectors.

## 4. Conclusions

This study focused on the coupling relationship among the near-surface state, N/P junction quality, and edge-related leakage current in planar HPGe detectors. A coupled process-optimization route consisting of low-damage near-surface construction, N/P junction formation, and guard-ring electrode regulation was established. CMP reduced the surface roughness Sa of the HPGe crystal to 6.68 nm and maintained good lattice integrity, providing a low-damage near-surface foundation for subsequent junction fabrication. On this basis, Li thermal diffusion and B ion implantation produced contact layers with favorable structural and electrical characteristics under the conditions of 0.5 Å s^−1^, 325 °C, and 5 min and Rp = 198.1 nm, respectively. After introducing the guard-ring electrode structure, the dark current of the central readout electrode decreased to 2.03 × 10^−9^ A at −20 V, and a stable switching current response was obtained under 12 keV monochromatic synchrotron X-ray irradiation. The Geant4 simulation results showed that the full-coverage electrode structure and the guard-ring electrode structure had only a small effect on the FEPE for ^55^Fe 5.9 keV low-energy X-rays, with FEPE values of 4.54% and 4.52%, respectively. Overall, this study demonstrates that the coupled optimization of near-surface treatment, N/P junction formation, and guard-ring electrode structure contributes to reducing the dark current of planar HPGe detectors and improving their current-response stability under 12 keV low-energy X-ray irradiation. Future work will further evaluate practical spectroscopic performance indicators, including energy resolution, peak-to-background ratio, charge-collection efficiency, and spectral stability, using a cryogenic low-noise spectroscopic readout system.

## Figures and Tables

**Table 1:** The 3D profilometry data of HPGe crystals under different polishing conditions.

Sample	Sa (nm)	PV (nm)	FWHM (nm)
80 r min^−1^–30 min–suede	86.24	788.32	191.28
80 r min^−1^–60 min–suede	85.49	636.87	153.68
100 r min^−1^–30 min–suede	80.79	777.11	150.19
100 r min^−1^–60 min–suede	70.20	537.97	109.51
80 r min^−1^–30 min–polyurethane	71.18	350.43	139.30
80 r min^−1^–60 min–polyurethane	61.53	326.4	135.76
100 r min^−1^–30 min–polyurethane	47.87	314.72	77.06
100 r min^−1^–60 min–polyurethane	46.01	309.66	96.96

**Table 2:** Room-temperature Hall measurement results of HPGe crystals at different Li thermal diffusion temperatures.

Li Diffusion Temperature (℃)	Bulk Carrier Concentration (cm^−3^)	Sheet Carrier Concentration (cm^−2^)	Resistivity (Ω·cm)	Mobility (cm^2^·V^−1^·s^−1^)
300	−1.37 × 10^14^	−1.78 × 10^13^	42.21	1082
325	−1.43 × 10^14^	−1.86 × 10^13^	37.50	1166
350	−1.63 × 10^14^	−2.12 × 10^13^	39.47	970

**Table 3:** Room-temperature Hall measurement results of HPGe crystals under different B-ion implantation conditions.

R_p_ (nm)	Bulk Carrier Concentration (cm^−3^)	Sheet Carrier Concentration (cm^−2^)	Resistivity (Ω·cm)	Mobility (cm^2^·V^−1^·s^−1^)
93.3	1.6 × 10^15^	2.07 × 10^14^	18.81	207.72
198.1	8.52 × 10^15^	4.26 × 10^14^	7.69	96.2
258.8	2.08 × 10^15^	2.7 × 10^14^	15.86	189.16

## Data Availability

The original contributions presented in this study are included in the article/Supplementary Materials. Further inquiries can be directed to the corresponding authors.

## Supplementary Materials

The following supporting information can be downloaded at: https://www.mdpi.com/article/10.3390/ma19143008/s1, Figure S1: Surface morphology of the HPGe crystal polished under the condition of 80 r/min–30 min–suede. Figure S2: Surface morphology of the HPGe crystal polished under the condition of 80 r/min–60 min–suede. Figure S3: Surface morphology of the HPGe crystal polished under the condition of 100 r/min–30 min–suede. Figure S4: Surface morphology of the HPGe crystal polished under the condition of 100 r/min–60 min–suede. Figure S5: Surface morphology of the HPGe crystal polished under the condition of 80 r/min–30 min–polyurethane. Figure S6: Surface morphology of the HPGe crystal polished under the condition of 80 r/min–60 min–polyurethane. Figure S7: Surface morphology of the HPGe crystal polished under the condition of 100 r/min–60 min–polyurethane. Figure S8: EDS mapping of the HPGe crystal surface. Table S1. Atomic percentages of elements on the HPGe crystal surface. Figure S9: XPS spectra of HPGe. Figure S10: Raman spectrum of HPGe. Figure S11: Low-temperature photoluminescence spectrum of HPGe at 77 K. Figure S12: Raman spectra of HPGe crystals treated at different Li deposition rates. Figure S13: XPS spectra of HPGe crystals treated at different Li deposition rates. Figure S14: XPS spectra of HPGe crystals diffused at different temperatures. Figure S15: XPS spectra of the HPGe crystal with an implantation depth of 93.3 nm before and after RTP. Figure S16: XPS spectra of the HPGe crystal with an implantation depth of 198.1 nm before and after RTP. Figure S17: XPS spectra of the HPGe crystal with an implantation depth of 258.8 nm before and after RTP. Figure S18: KPFM image of HPGe after B ion implantation. Figure S19: Low-temperature photoluminescence spectrum of HPGe after B ion implantation at 77 K.
