# Supplementary material for: Junction Formation and Leakage Current Suppression in Planar High-Purity Germanium Detectors for Low-Energy X-Ray Detection
Source: Materials (Basel). 2026 Jul 13;19(14):3008. doi: 10.3390/ma19143008 (PMC13412493; doi:10.3390/ma19143008)
Supplement: Supplementary file 1 [file materials-19-03008-s001.zip › materials-4382368-supplementary.pdf]

Junction Formation and Leakage Current Suppression in Planar High-Purity Germanium Detectors for Low-Energy X-ray Detection

Meng Cao[1,2,*], Qingzhi Hu[1,†], Yanggang Jia[1,†], Zexin Wang[1], Zhaoran Guan[1], Haofei Huang[1,*], Linjun Wang[1,2,3,*], Jian Huang[1,2,3]

[1] State Key Laboratory of Materials for Advanced Nuclear Energy & School of Materials Science and Engineering, Shanghai University, Shanghai 200444, China

[2] Zhejiang Institute of Advanced Materials, SHU, Jiashan, 314113, China

[3] Shanghai Engineering Research Center for Integrated Circuits and Advanced Display Materials, Shanghai, China

* To whom correspondence should be addressed:

Corresponding author: Meng Cao; Haofei Huang; Linjun Wang

*E-mail address: caomeng@shu.edu.cn;

kardson@shu.edu.cn

ljwang@shu.edu.cn

[†]: Qingzhi Hu and Yanggang Jia contributed equally to this work.

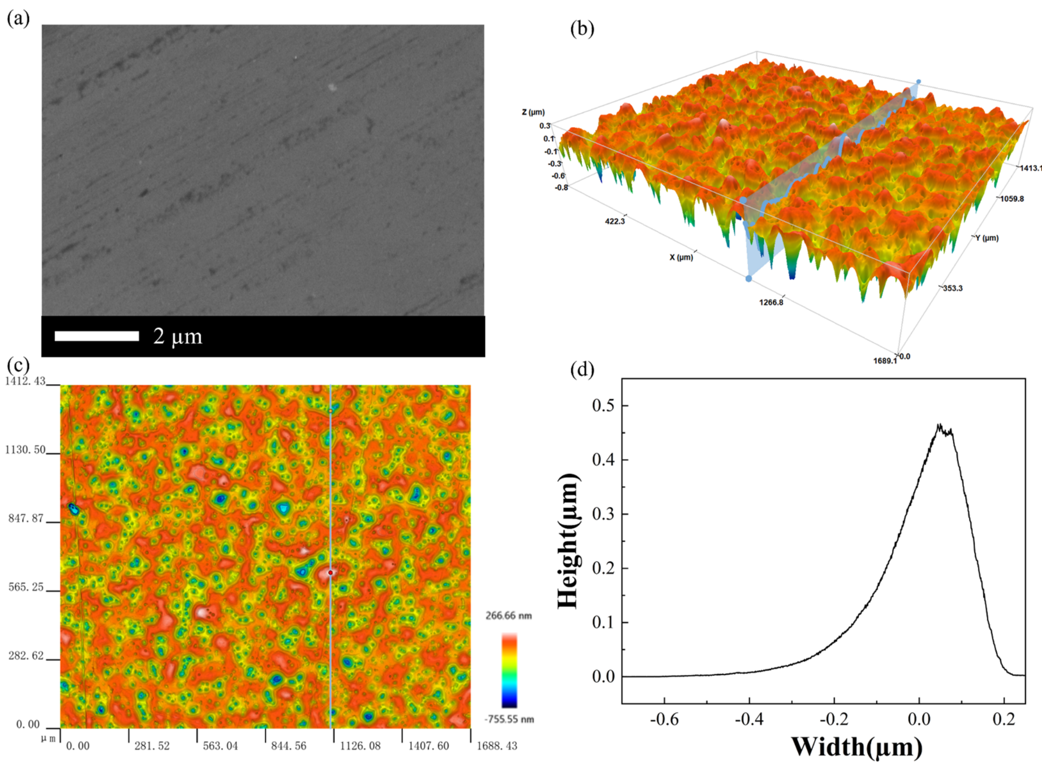

Figure S1. Surface morphology of the HPGe crystal polished under the condition of 80 r/min–30 min–suede: (a) SEM image; (b) 3D topographic map and height distribution; (c) 2D surface map; (d) roughness distribution histogram.

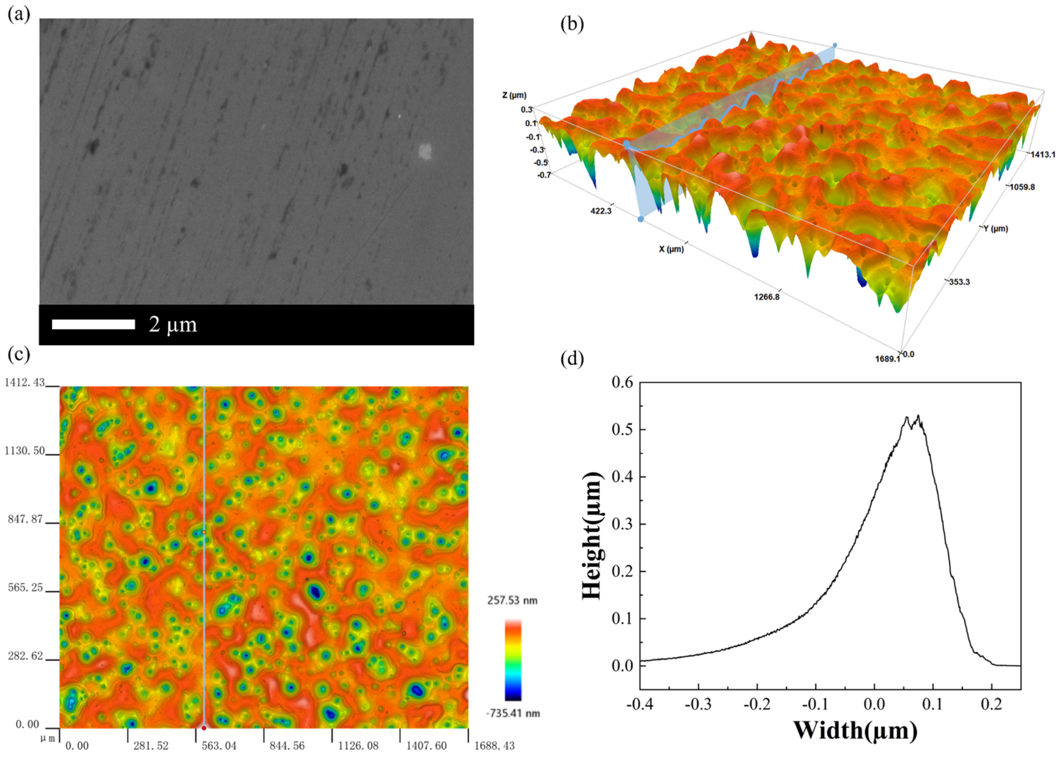

Figure S2. Surface morphology of the HPGe crystal polished under the condition of 80 r/min–60 min–suede: (a) SEM image; (b) 3D topographic map and height distribution; (c) 2D surface map; (d) roughness distribution histogram.

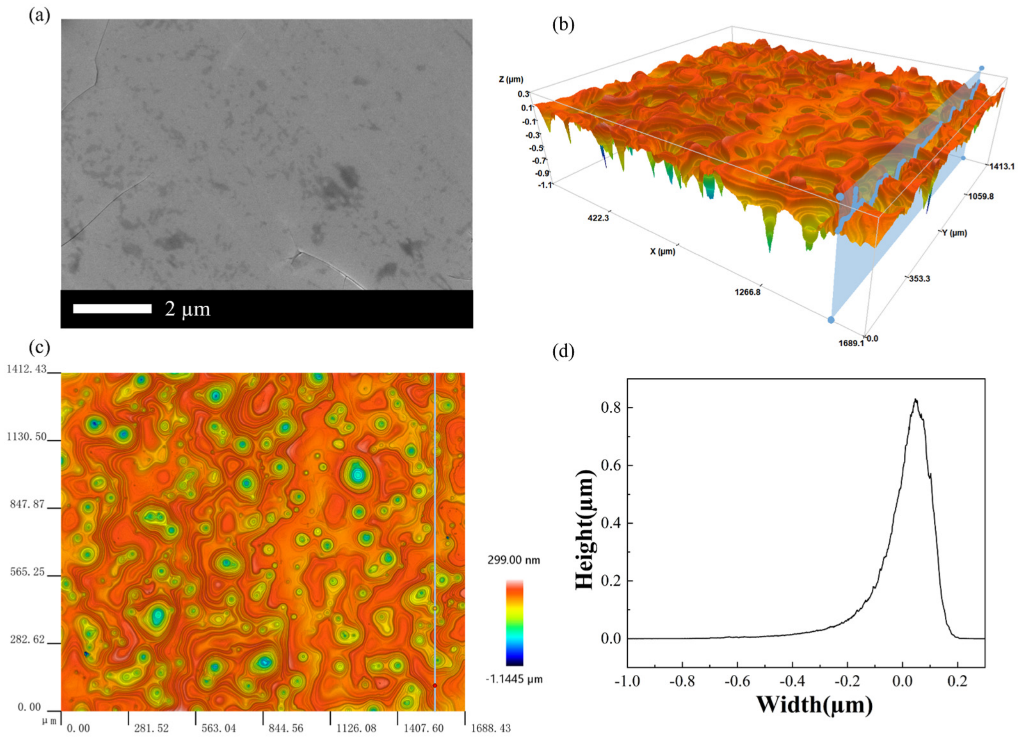

Figure S3. Surface morphology of the HPGe crystal polished under the condition of 100 r/min–30 min–suede: (a) SEM image; (b) 3D topographic map and height distribution; (c) 2D surface map; (d) roughness distribution histogram.

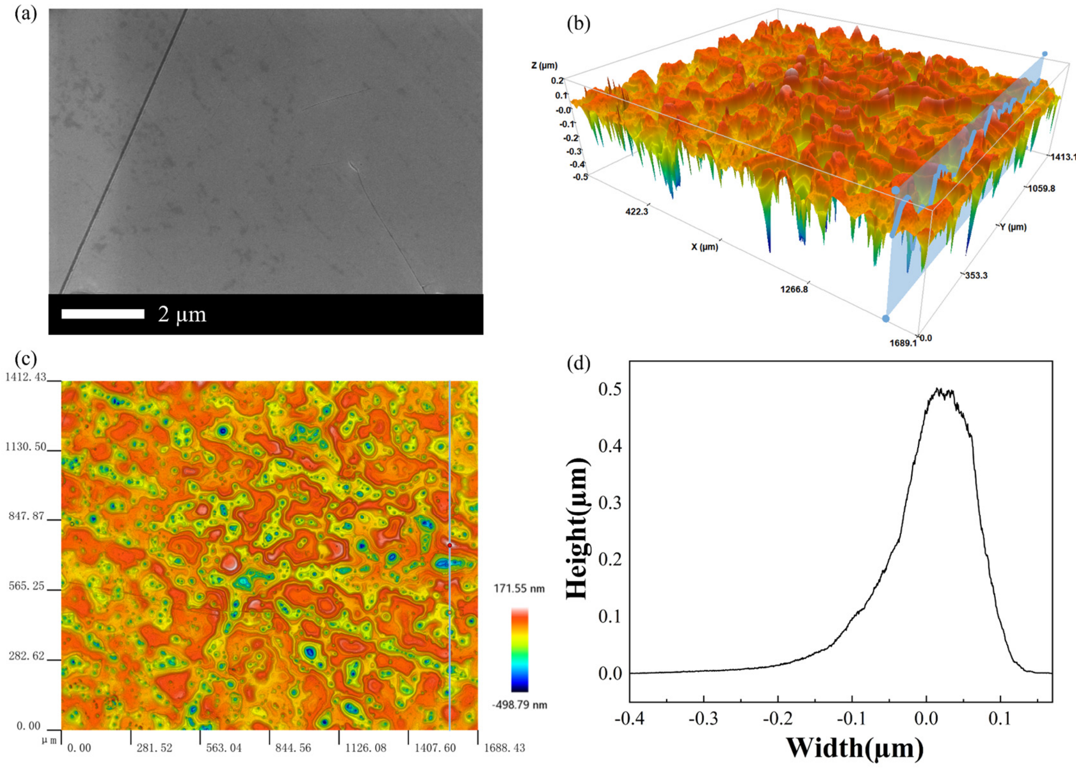

Figure S4. Surface morphology of the HPGe crystal polished under the condition of 100 r/min–60 min–suede: (a) SEM image; (b) 3D topographic map and height distribution; (c) 2D surface map; (d) roughness distribution histogram.

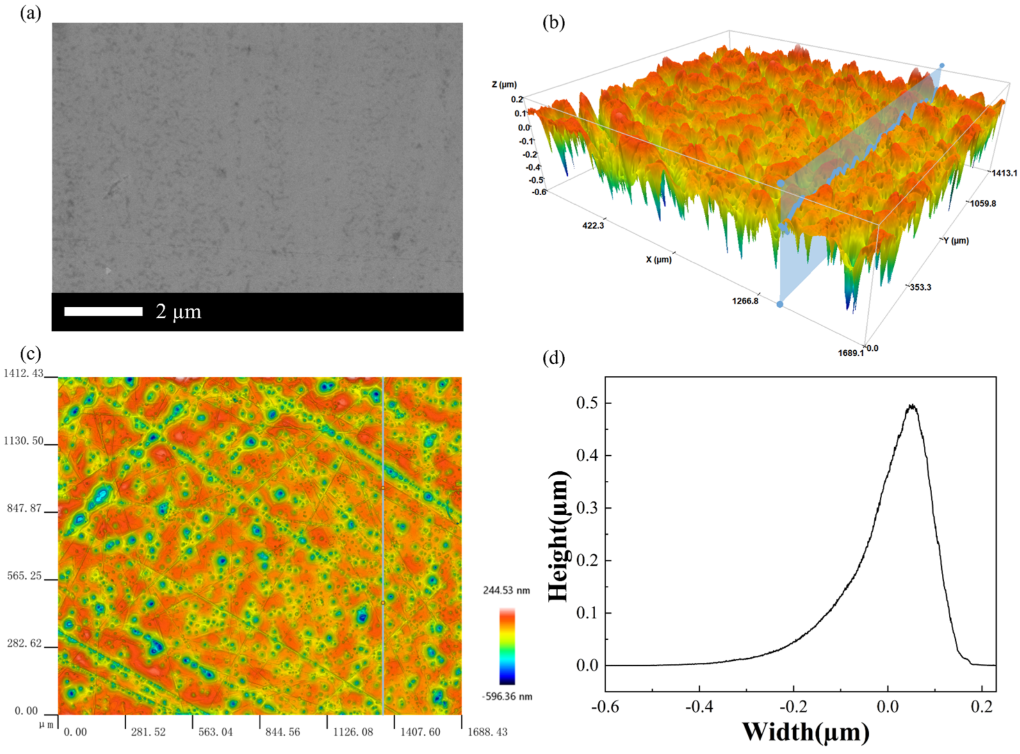

Figure S5. Surface morphology of the HPGe crystal polished under the condition of 80 r/min–30 min–polyurethane: (a) SEM image; (b) 3D topographic map and height distribution; (c) 2D surface map; (d) roughness distribution histogram.

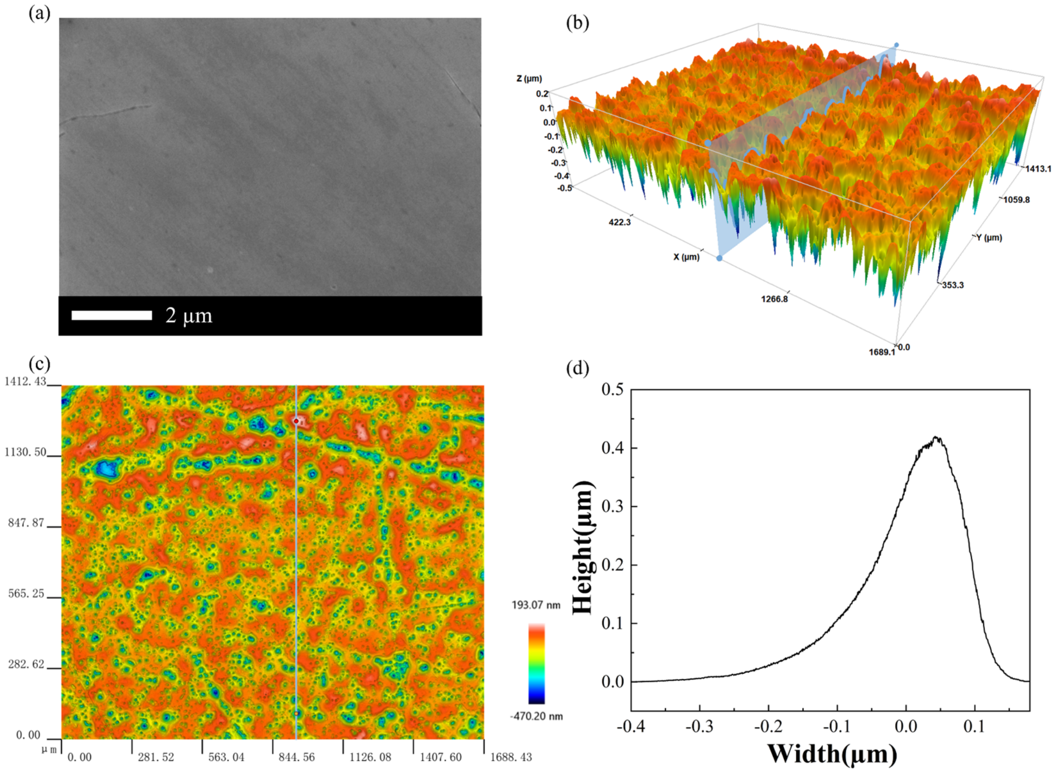

Figure S6. Surface morphology of the HPGe crystal polished under the condition of 80 r/min–60 min–polyurethane: (a) SEM image; (b) 3D topographic map and height distribution; (c) 2D surface map; (d) roughness distribution histogram.

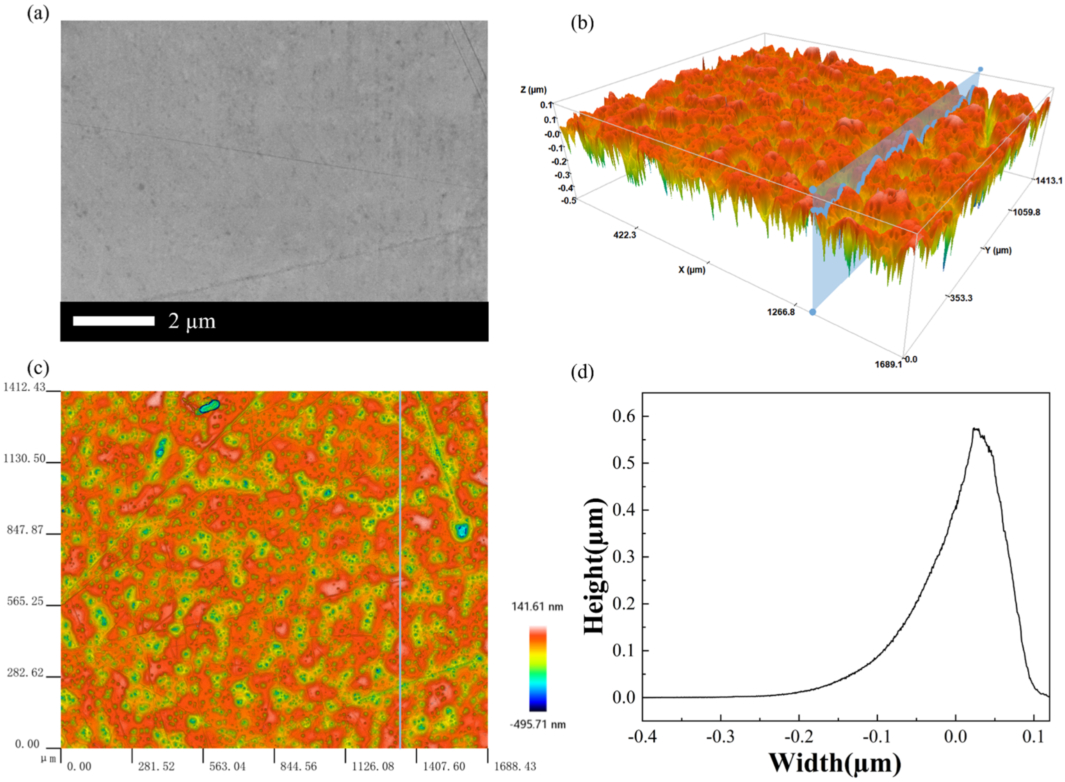

Figure S7. Surface morphology of the HPGe crystal polished under the condition of 100 r/min–60 min–polyurethane: (a) SEM image; (b) 3D topographic map and height distribution; (c) 2D surface map; (d) roughness distribution histogram.

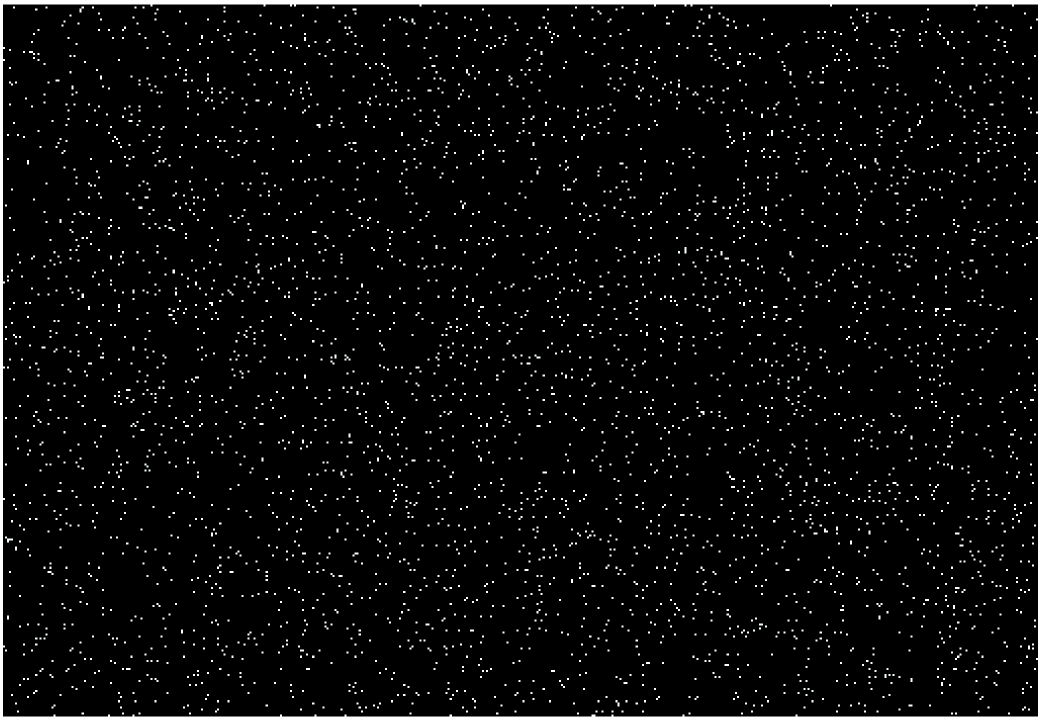

Figure S8. EDS mapping of the HPGe crystal surface.

Table. S1. Atomic percentages of elements on the HPGe crystal surface.

Sample	Ge (%)	O (%)
HPGe	99.14	0.86

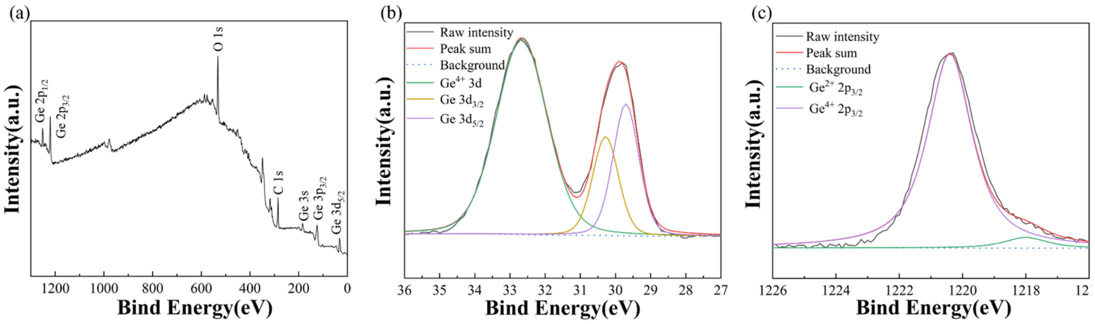

Figure S9. XPS spectra of HPGe: (a) survey spectrum; (b) Ge 3d core-level spectrum; (c) Ge 2p core-level spectrum.

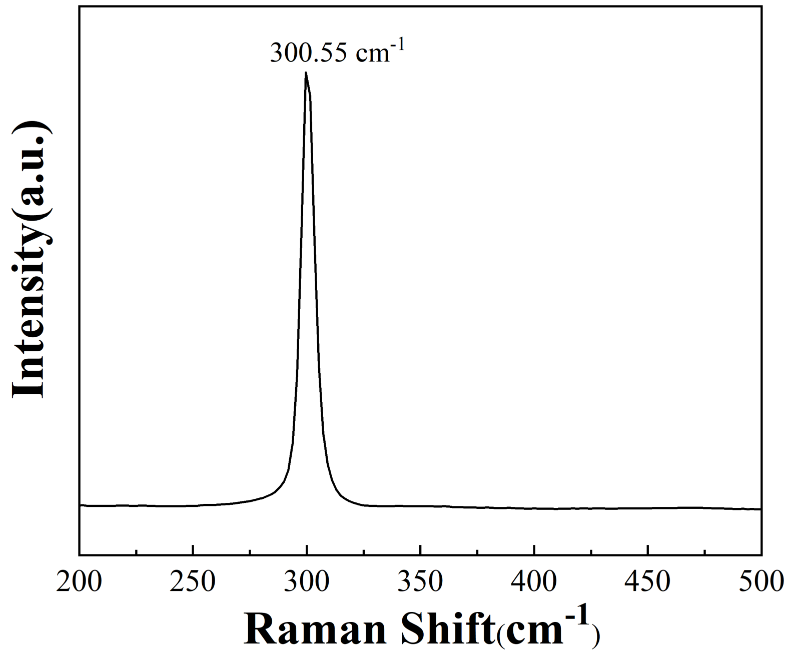

Figure S10. Raman spectrum of HPGe.

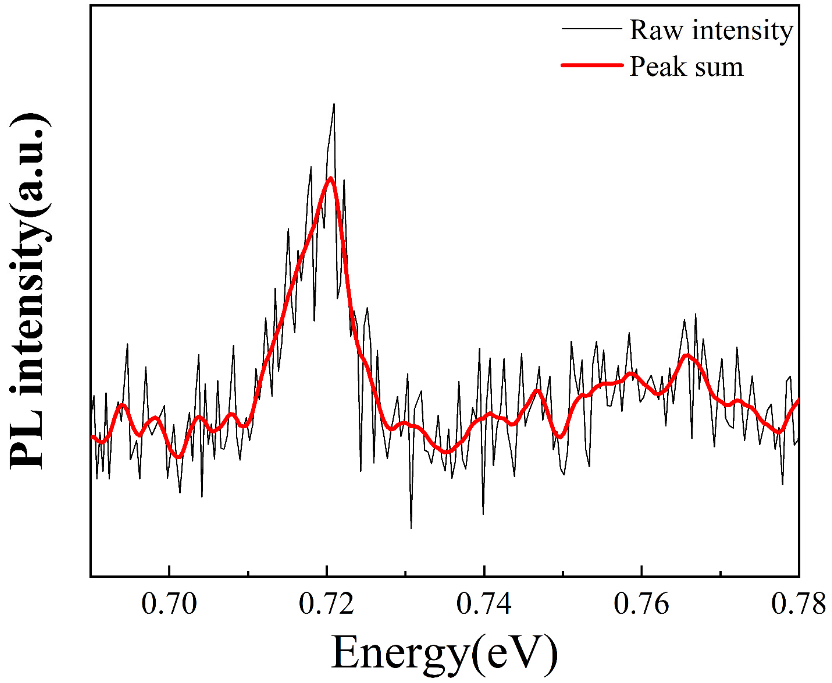

Figure S11. Low-temperature photoluminescence spectrum of HPGe at 77 K.

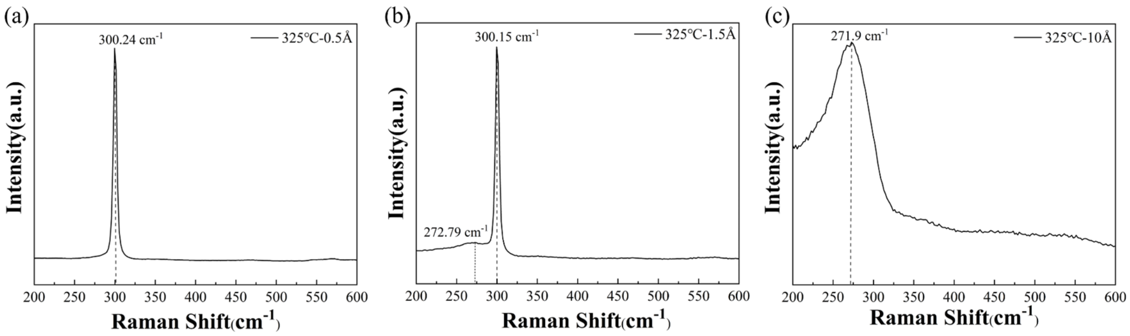

Figure S12. Raman spectra of HPGe crystals treated at different Li deposition rates: (a) 0.5 Å/s; (b) 1.5 Å/s; (c) 10 Å/s.

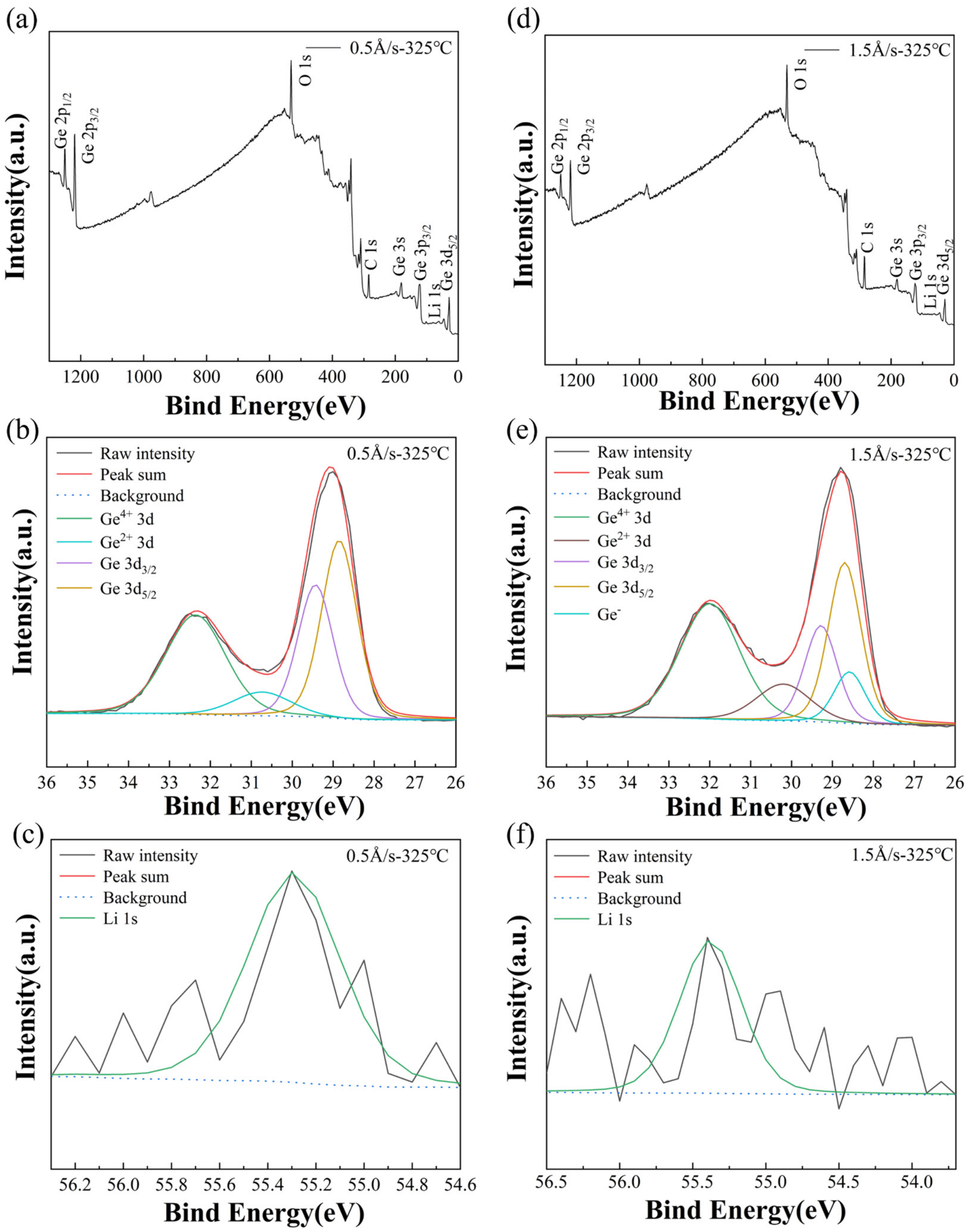

Figure S13. XPS spectra of HPGe crystals treated at different Li deposition rates: (a) survey spectrum at 0.5 Å/s; (b) Ge 3d core-level spectrum at 0.5 Å/s; (c) Li 1s core-level spectrum at 0.5 Å/s; (d) survey spectrum at 1.5 Å/s; (e) Ge 3d core-level spectrum at 1.5 Å/s; (f) Li 1s core-level spectrum at 1.5 Å/s.

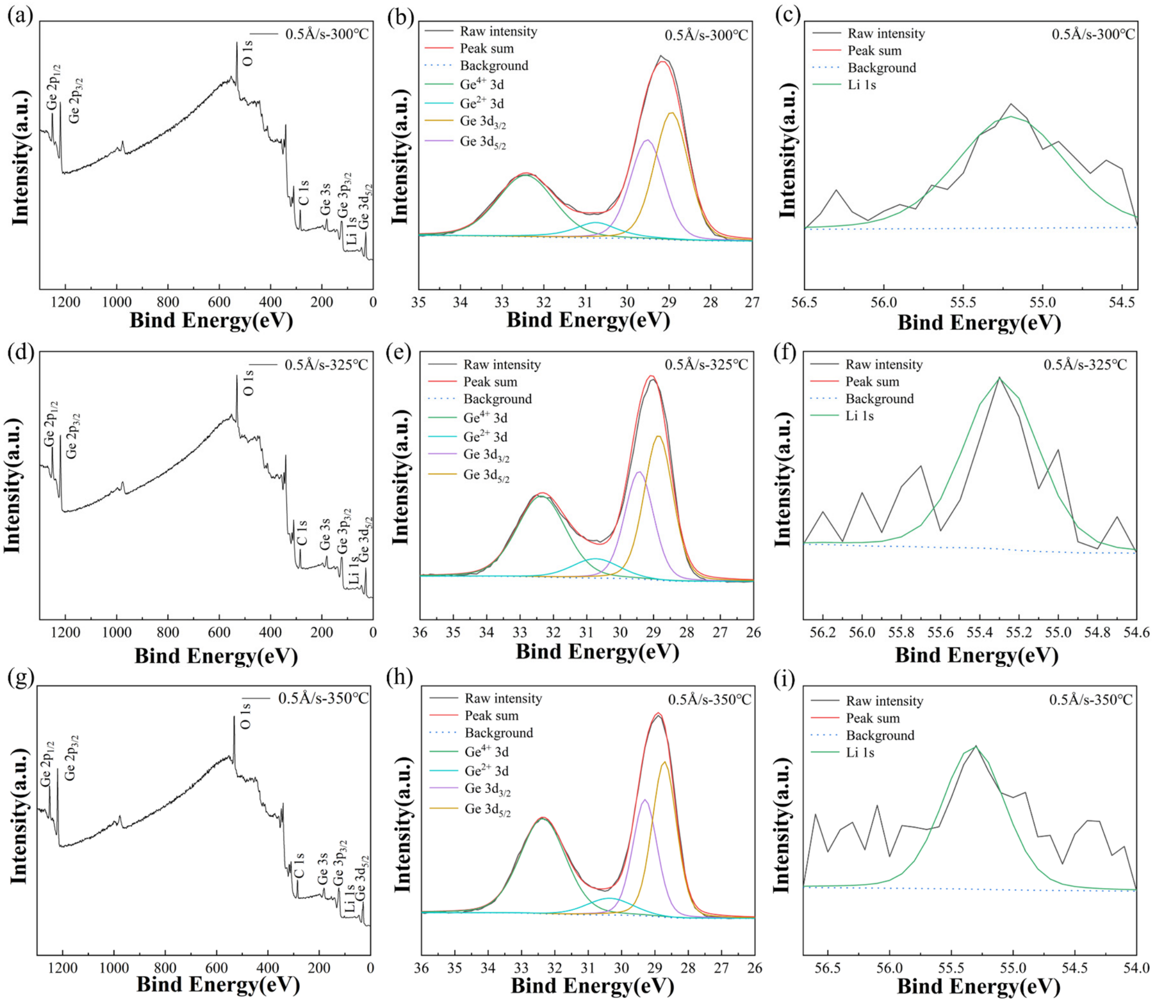

Figure S14. XPS spectra of HPGe crystals diffused at different temperatures: (a) survey spectrum at 300 °C; (b) Ge 3d core-level spectrum at 300 °C; (c) Li 1s core-level spectrum at 300 °C; (d) survey spectrum at 325 °C; (e) Ge 3d core-level spectrum at 325 °C; (f) Li 1s core-level spectrum at 325 °C; (g) survey spectrum at 350 °C; (h) Ge 3d core-level spectrum at 350 °C; (i) Li 1s core-level spectrum at 350 °C.

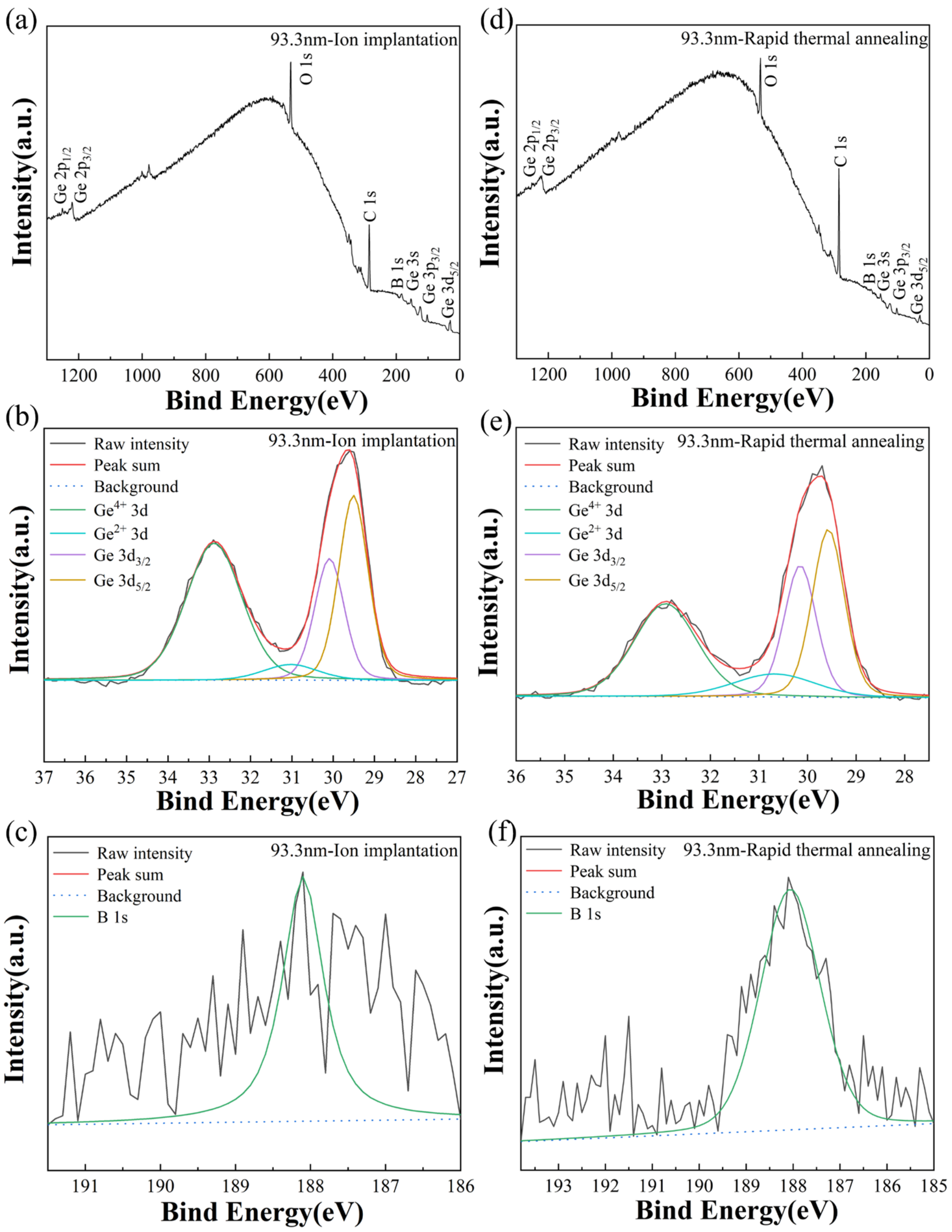

Figure S15. XPS spectra of the HPGe crystal with an implantation depth of 93.3 nm before and after RTP: (a) survey spectrum before RTP; (b) Ge 3d core-level spectrum before RTP; (c) B 1s core-level spectrum before RTP; (d) survey spectrum after RTP; (e) Ge 3d core-level spectrum after RTP; (f) B 1s core-level spectrum after RTP.

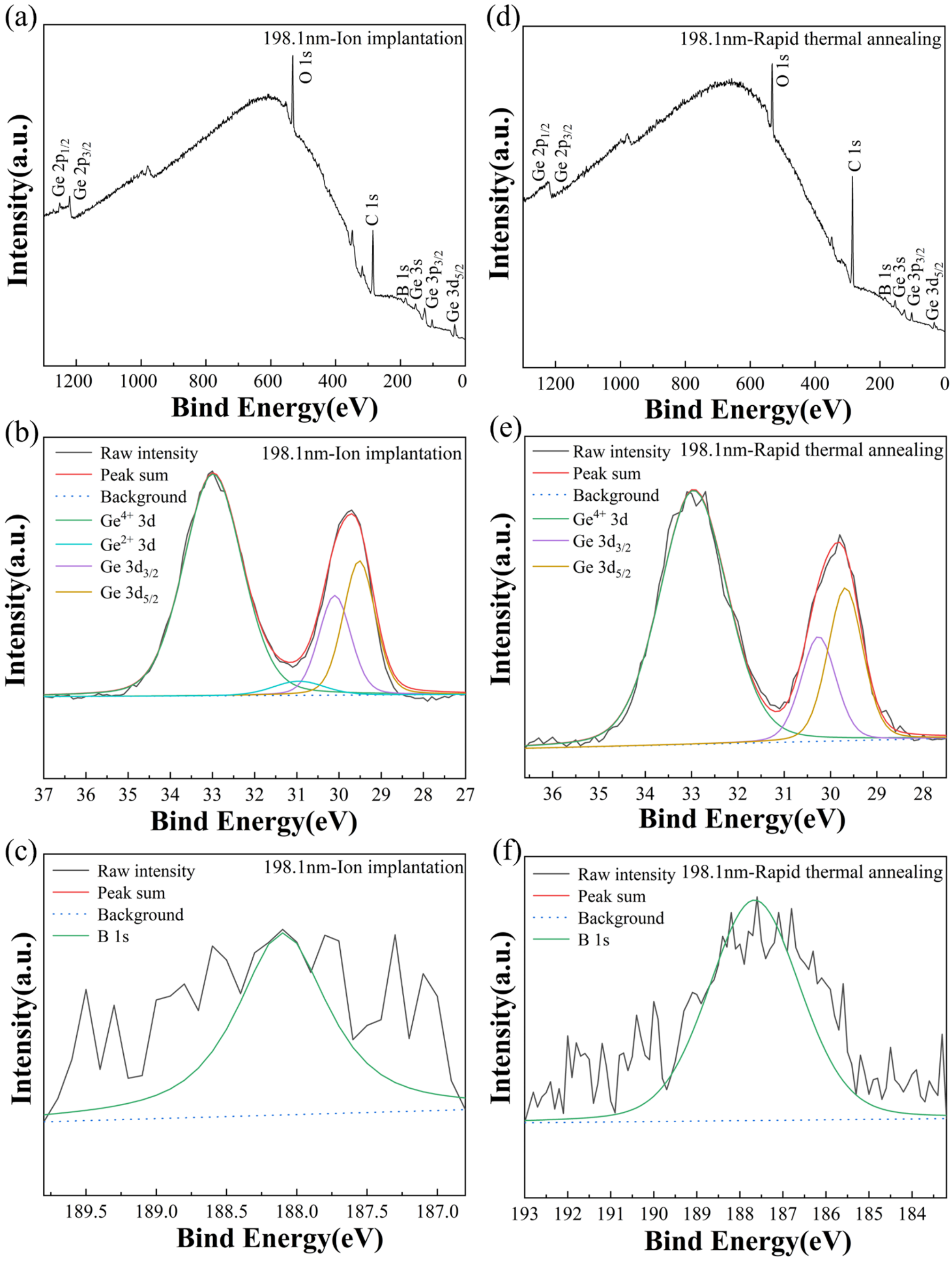

Figure S16. XPS spectra of the HPGe crystal with an implantation depth of 198.1 nm before and after RTP: (a) survey spectrum before RTP; (b) Ge 3d core-level spectrum before RTP; (c) B 1s core-level spectrum before RTP; (d) survey spectrum after RTP; (e) Ge 3d core-level spectrum after RTP; (f) B 1s core-level spectrum after RTP.

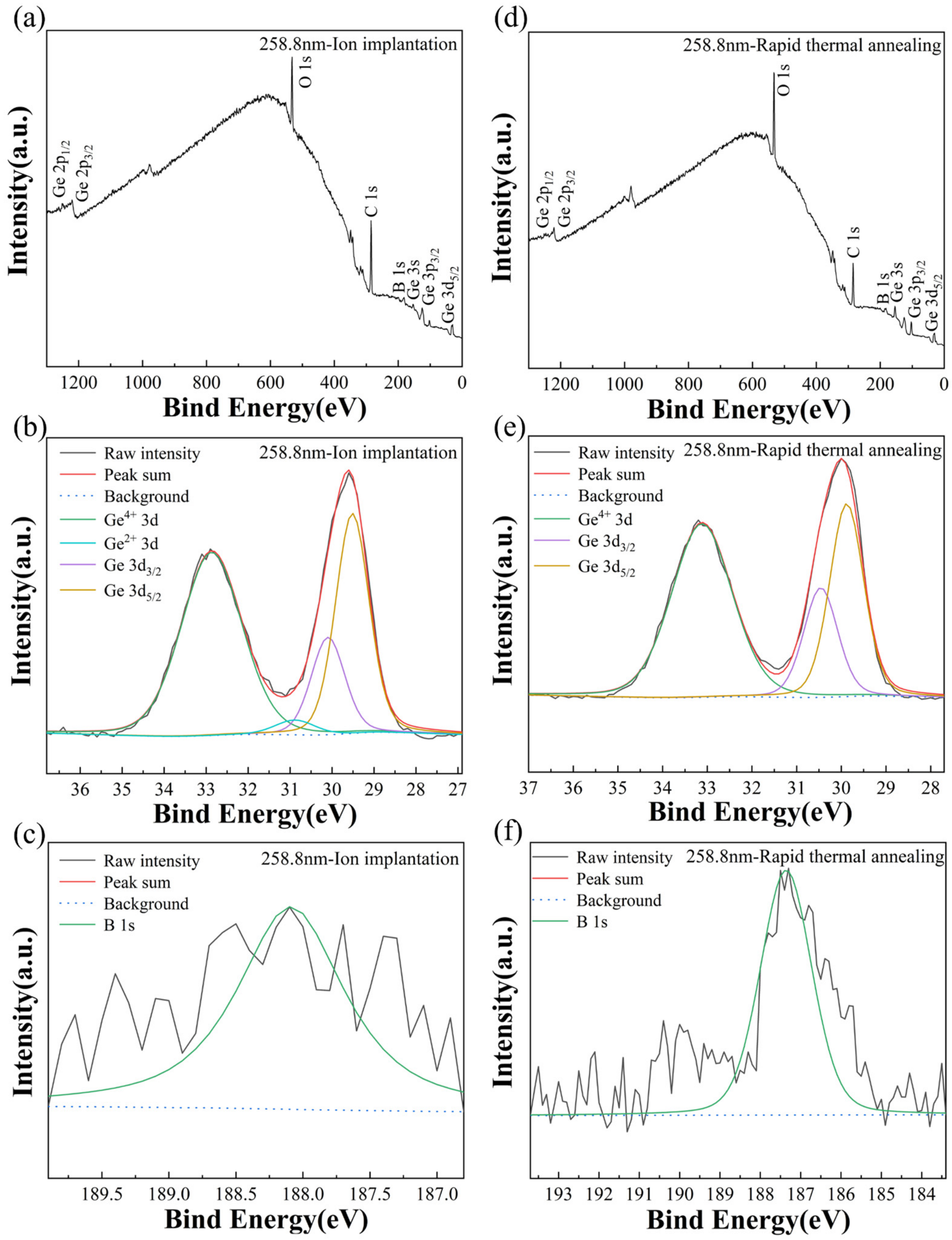

Figure S17. XPS spectra of the HPGe crystal with an implantation depth of 258.8 nm before and after RTP: (a) survey spectrum before RTP; (b) Ge 3d core-level spectrum before RTP; (c) B 1s core-level spectrum before RTP; (d) survey spectrum after RTP; (e) Ge 3d core-level spectrum after RTP; (f) B 1s core-level spectrum after RTP.

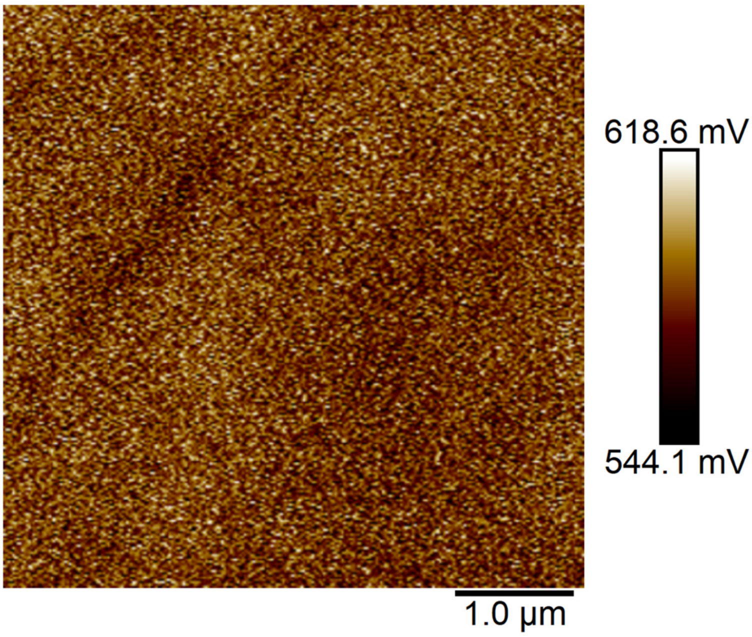

Figure S18. KPFM image of HPGe after B ion implantation.

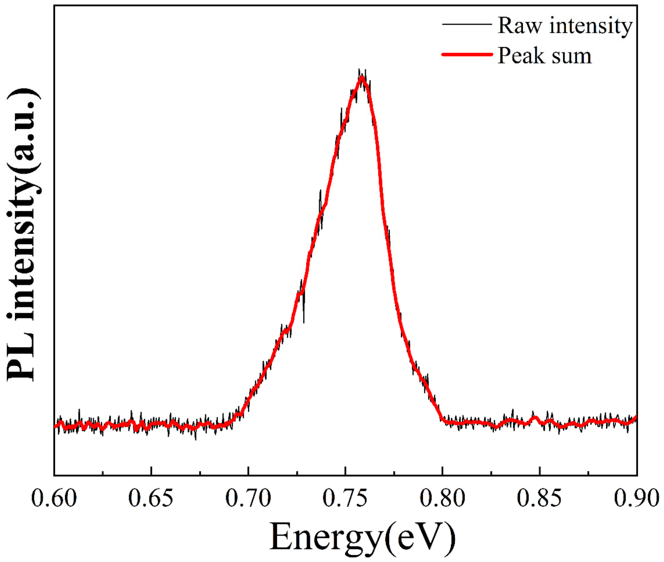

Figure S19. Low-temperature photoluminescence spectrum of HPGe after B ion implantation at 77 K.
